# Interpositional Arthroplasty With Quadriceps Tendon in Patellofemoral Joint Osteoarthritis: An Alternative Way

**DOI:** 10.7759/cureus.43920

**Published:** 2023-08-22

**Authors:** Muhammad Ashraf Fareeq Shamsuddin, Khairil Anwar Ahmad Hanif, Mohd Nizlan Mohd Nasir

**Affiliations:** 1 Orthopaedics and Traumatology, Hospital Pengajar Universiti Putra Malaysia, Serdang, MYS; 2 Sports Surgery Division, Universiti Putra Malaysia, Serdang, MYS

**Keywords:** osteoarthritis, orthopaedics, interpositional arthroplasty, patellofemoral joint osteoarthritis, arthroplasty

## Abstract

We present a case report of a 45-year-old Malay female prison officer with a diagnosis of lateral patellofemoral joint (PFJ) osteoarthritis (OA) in her right knee for whom conservative treatment failed. She was periodically followed up for the unresolving anterior right knee pain, and the patient was offered interpositional PFJ arthroplasty with the quadriceps tendon.

A novel technique of interpositional PFJ arthroplasty using lateral inner section ipsilateral quadriceps tendon was applied. The approach and surgical technique were described in this case report. The aim of this study is to describe why this technique was chosen, step by step with images on how interpositional PFJ arthroplasty is done and its satisfactory outcome following a three-month follow up.

## Introduction

The incidence of osteoarthritis (OA) among younger people is increasing, and there is no clear consensus on the treatment of the early onset of this condition. A recent 2021 study found that 30.4% of the 4565 cases reviewed were diagnosed before the age of 45 years [1]. OA can impact all three compartments of the knee, and the patellofemoral joint (PFJ) is one of the most frequently occurring compartments that causes knee pain despite normal radiographical findings [2]. According to a study conducted in 2006, it was found that 24% of diagnosed knee OA cases had only unicompartmental PFJ OA as detected through radiographical studies done by rheumatologists [3].

The treatment for isolated PFJ arthritis is not well documented in the available literature and textbooks. Most of the literature discusses the management of a homogenous group with three compartments, and there is little research on the treatment for PFJ arthritis. Conservative management techniques, such as patella tapping [4] and knee brace usage [5], have been documented, but these methods did not work for this particular patient. A partial lateral patellectomy was done on our patient, who is young and active with isolated unicompartmental PFJ OA [6] and conservative management failed for this patient. This was when the idea of interpositional arthroplasty came into the picture. This case study highlights and explains the step-by-step methods used in interpositional arthroplasty and the results of the surgical procedure.

## Case presentation

**Figure 1:**
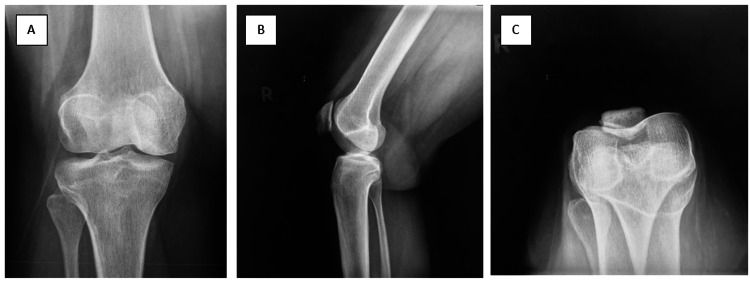
Images A to C showing radiographs of the patient's right knee in AP, lateral, and Merchant's views. AP: Anteroposterior

A 45-year-old Malay female patient, who works as a prison officer, is under our orthopedic follow-up for isolated anterior right knee pain for the past five years. The patient described the pain as mechanical pain which worsens as she walks, which is associated with crepitus and limited range of motion in flexion. Pain score ranged 3-5 over 10 according to the visual analog pain score. She was followed up in a different hospital before her presentation to us. A diagnosis of lateral facet PFJ arthroplasty was made. She underwent multiple conservative management with analgesia and physiotherapy but failed. She was then done lateral patella-facetectomy in 2016. However, the outcome of the procedure was not satisfactory as the patient still had anterior right knee pain. Our clinical examination revealed lateral patella facet and lateral joint line tenderness. The tenderness does not affect the range of motion of the knee. No ligamentous laxity or meniscal injury was elicited during the examination. The patient was walking with an antalgic gait over her right lower limb. All blood investigations taken showed nonsuggestive inflammatory arthritis or auto-immune disease. All previous radiographical studies were retrieved and reviewed to understand the unresolved anterior right knee pain. Figure 1 shows the plain radiograph of the patient's right knee taken before the operation. 

**Figure 2:**
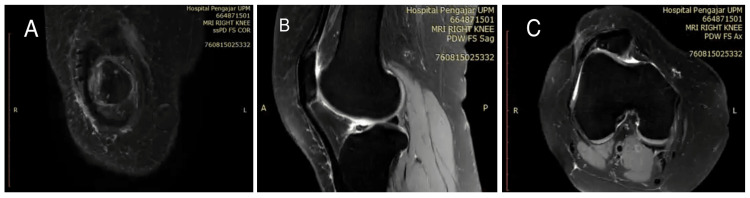
Images A to C showing MRI PDW-FS of right knee in coronal, sagittal, and axial cuts. MRI: Magnetic resonance imaging; PDW-FS: proton-density weighted in fat suppressed

A magnetic resonance imaging (MRI) study of the right knee done revealed thinning of lateral patella facet (postlateral facetectomy) with normal cruciate ligament and meniscus. Figure 2 shows the MRI of right knee proton-density weighted in fat suppressed (PDW-FS) in coronal, sagittal, and axial cuts.

The patient was then scheduled for a diagnostic arthroscopy and debridement of the right knee in June 2022. Arthroscopic findings include attenuated cartilage at the lateral facet of the patella and outerbridge 2 medial patella facet chondral injury with normal cruciate ligament and meniscus. Given the patient has done lateral facetectomy and the patella thickness is 12mm, hence, the option of patella resurfacing is not favorable. Thus, the patient was counseled for interpositional PFJ arthroplasty with the quadriceps tendon.

**Figure 3:**
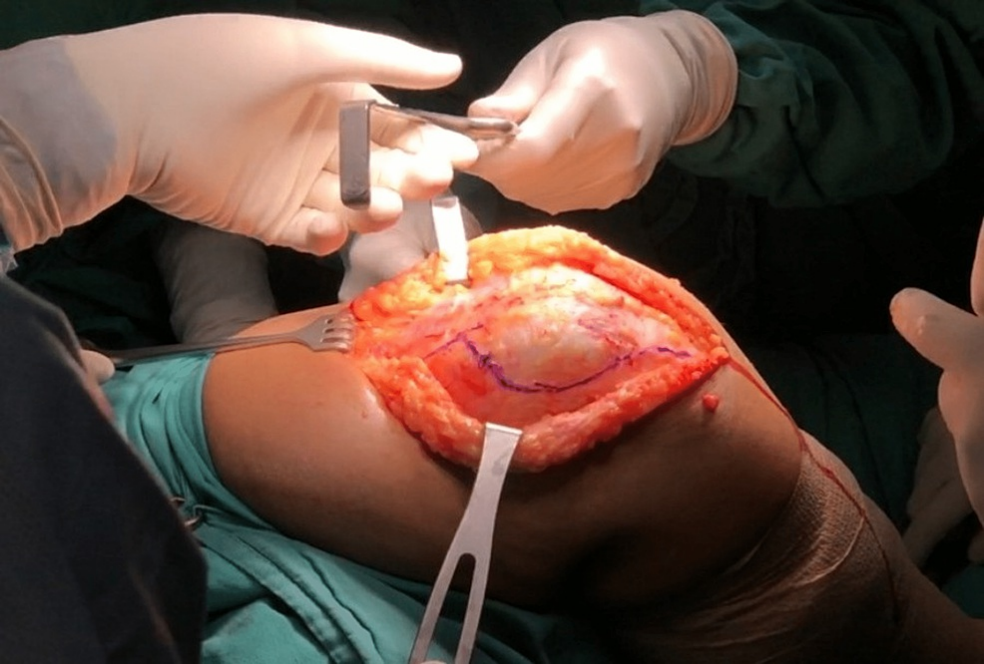
The lateral parapatellar approach was done to harvest the inner third lateral section of the ipsilateral quadriceps tendon.

**Figure 4:**
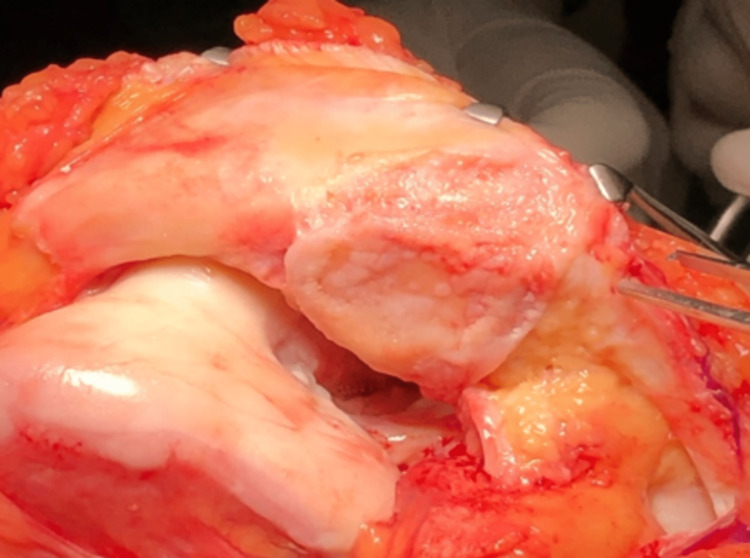
The patella was flipped medially to expose both the medial and lateral facet and site of the quadriceps tendon to be harvested.

**Figure 5:**
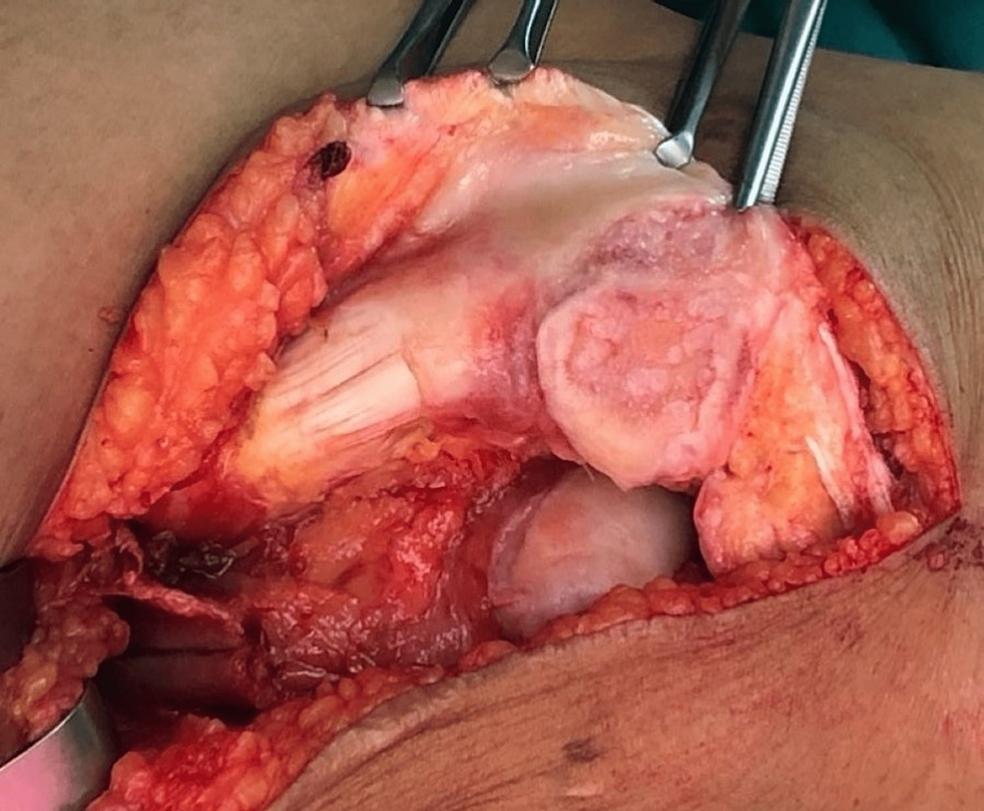
The width and length of the chondral defect of the medial and lateral patella facet were measured.

**Figure 6:**
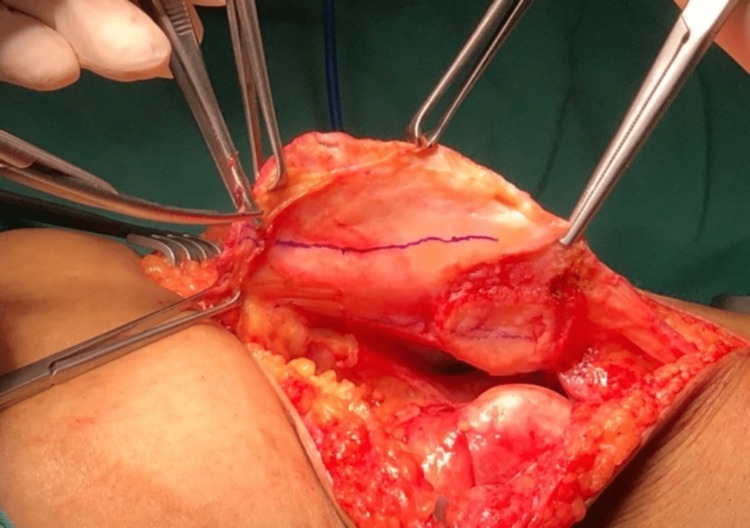
The quadriceps tendon is harvested according to the size of the defect.

**Figure 7:**
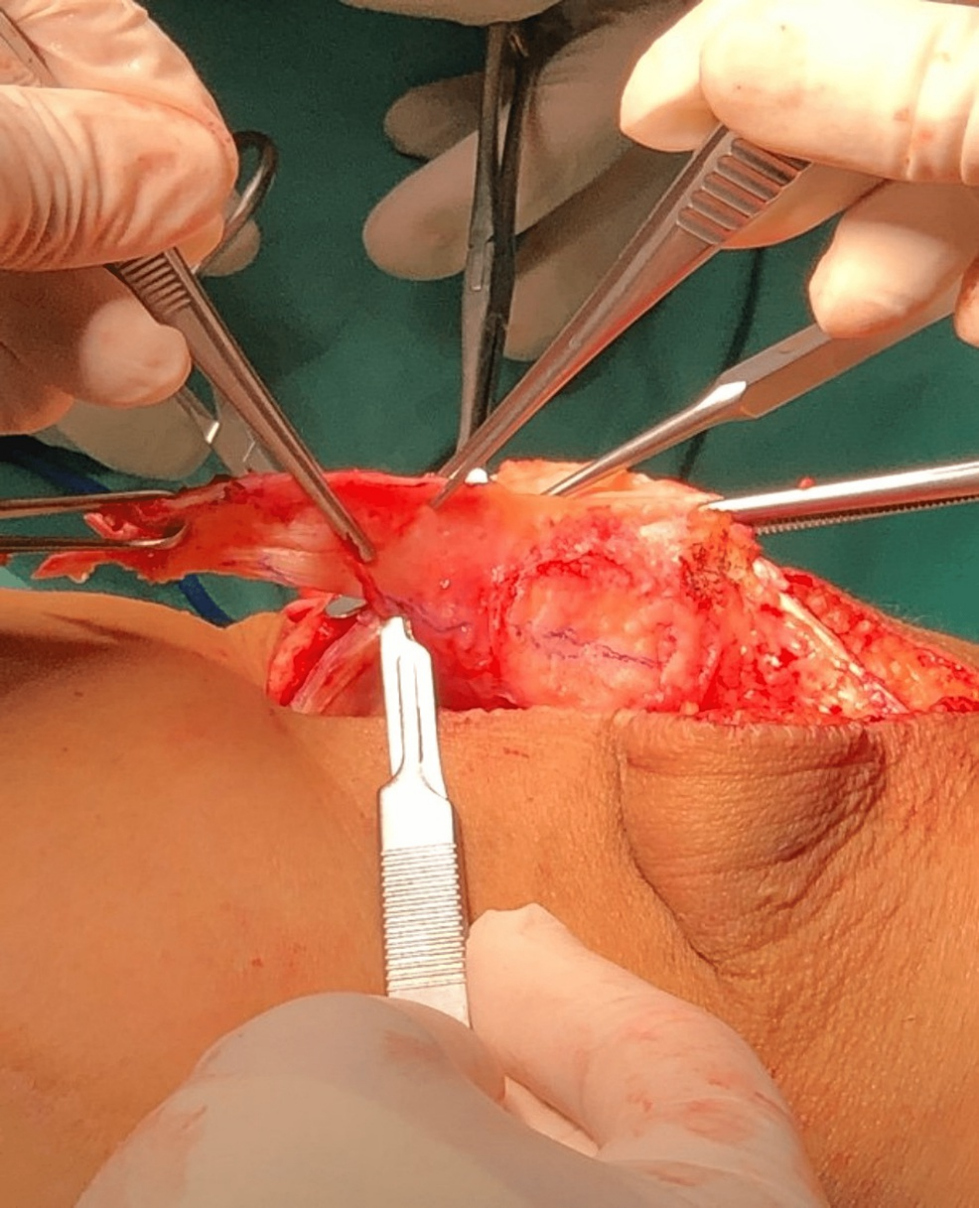
The tendon was harvested from proximal to distal as the attachment of the quadriceps tendon on the superior pole of the patella is preserved.

**Figure 8:**
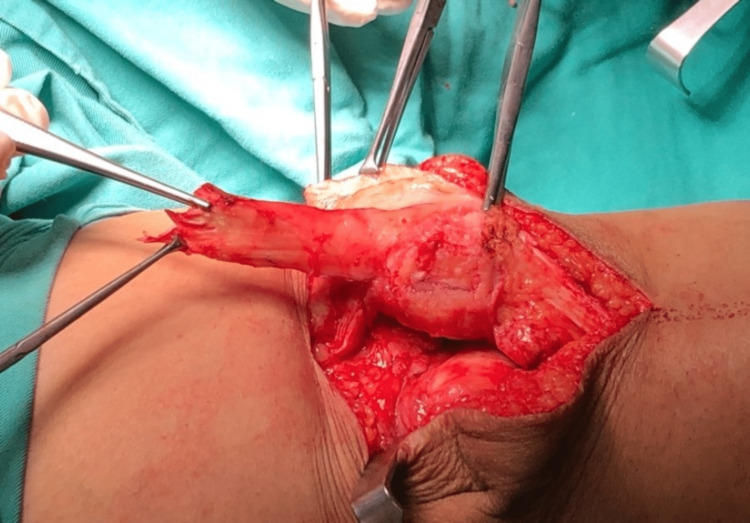
The harvested quadriceps tendon was then layered using a 15-size scalpel to reach 15mm thickness. This is to avoid overstuffing in the patellofemoral joint.

**Figure 9:**
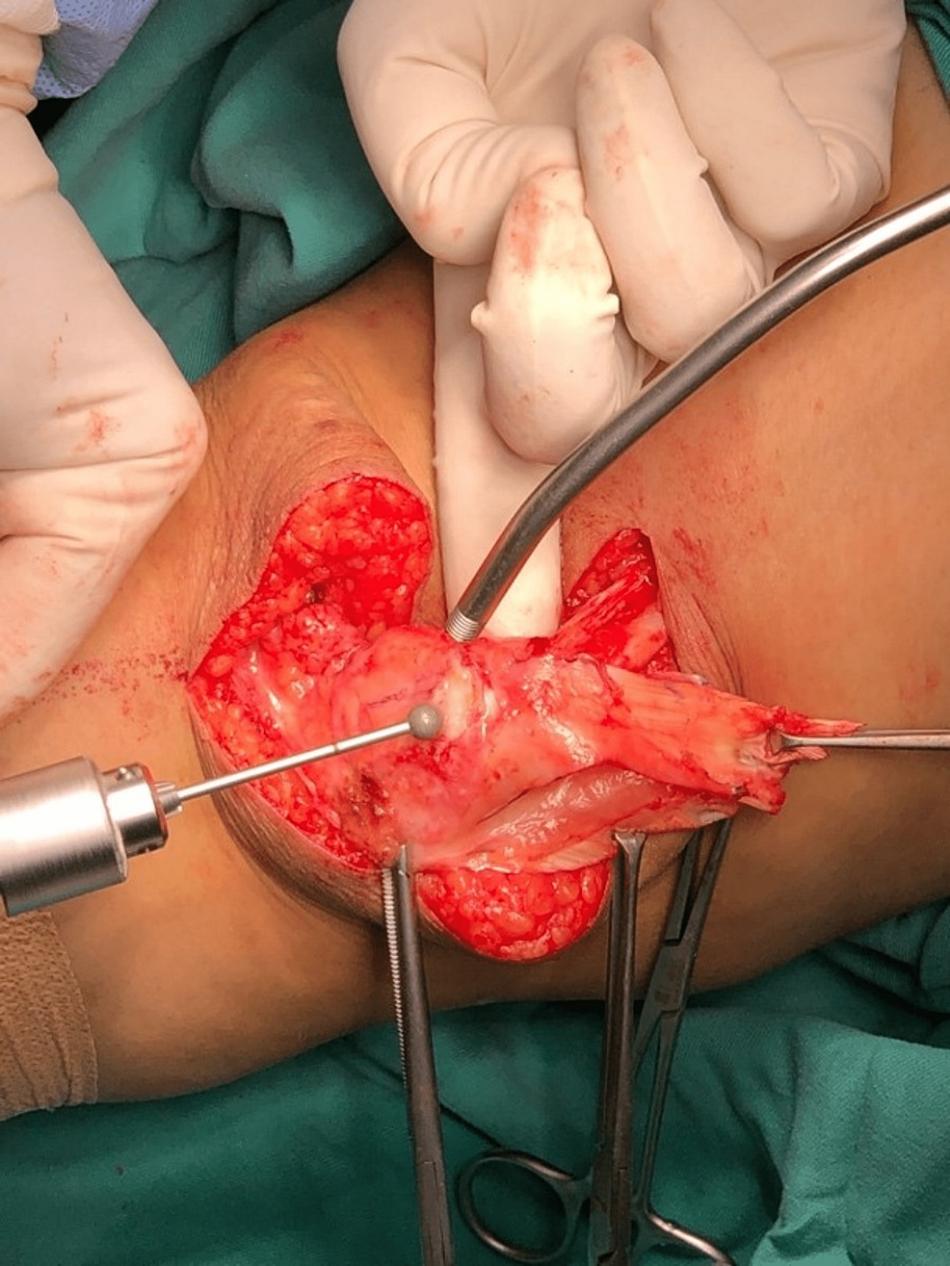
The lateral and medial facet of the patella was then minimally resurfaced using a bone burr.

**Figure 10:**
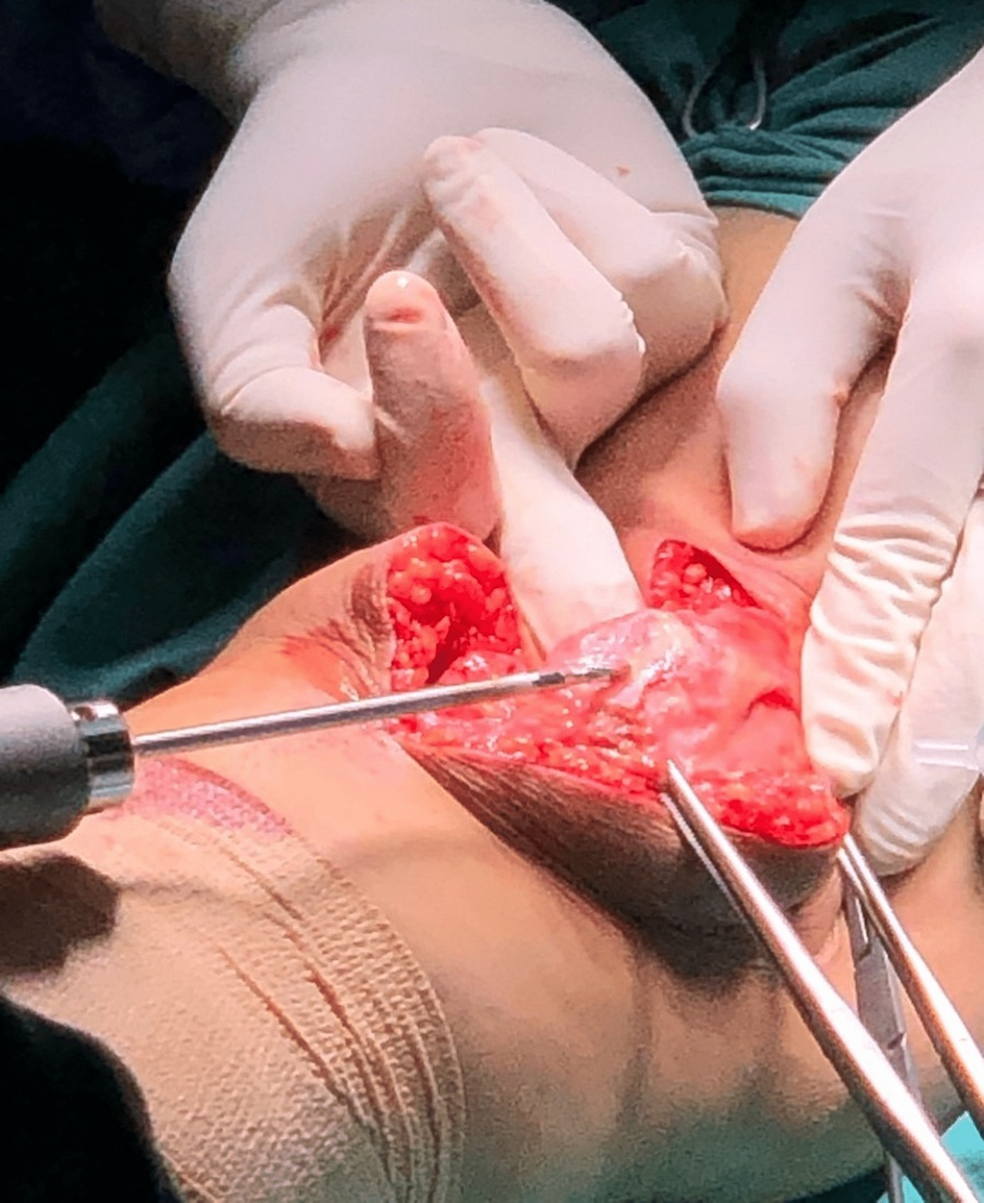
Four holes were made on the patella with a size 2.5mm drill bit to anchor the Ethibond suture.

**Figure 11:**
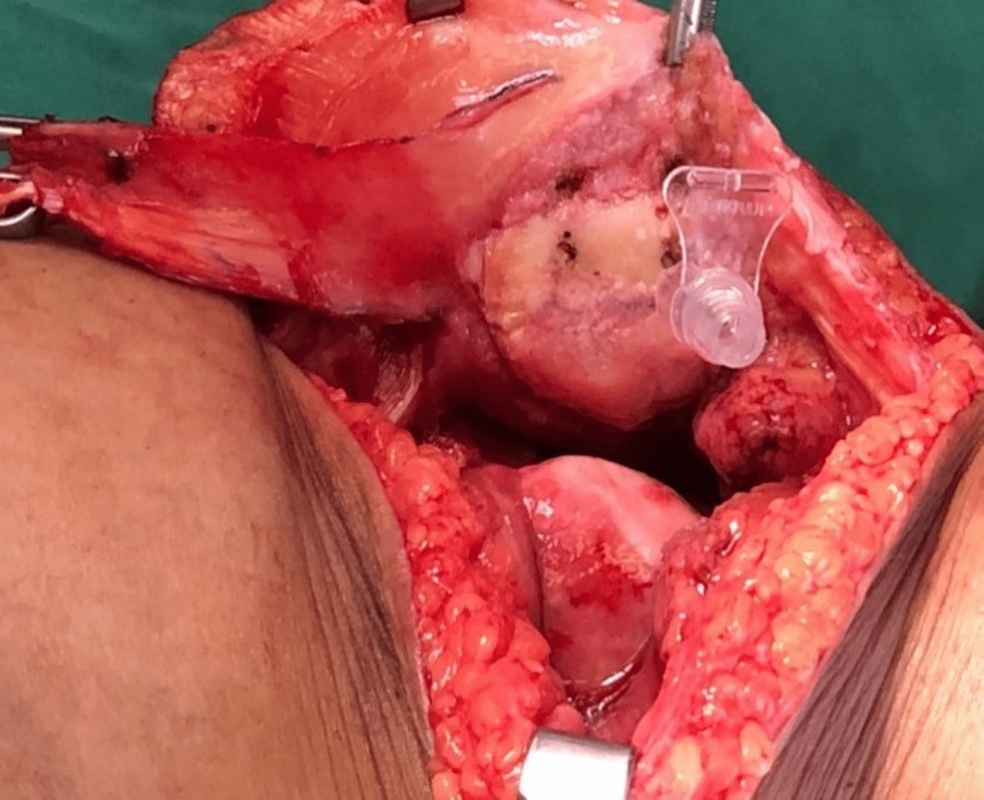
A cannula was used to pass the Ethibond suture through the drilled hole.

**Figure 12:**
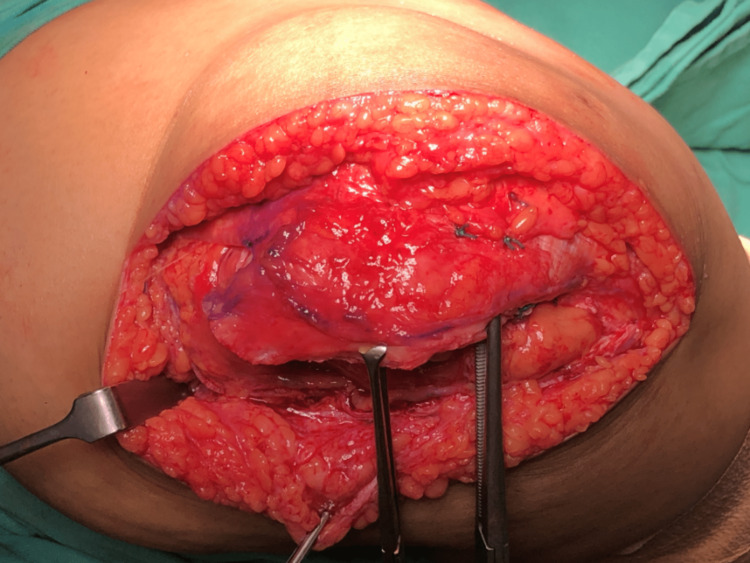
Using the Ethibond suture, the harvested quadriceps tendon was sutured to the posterior patella.

**Figure 13:**
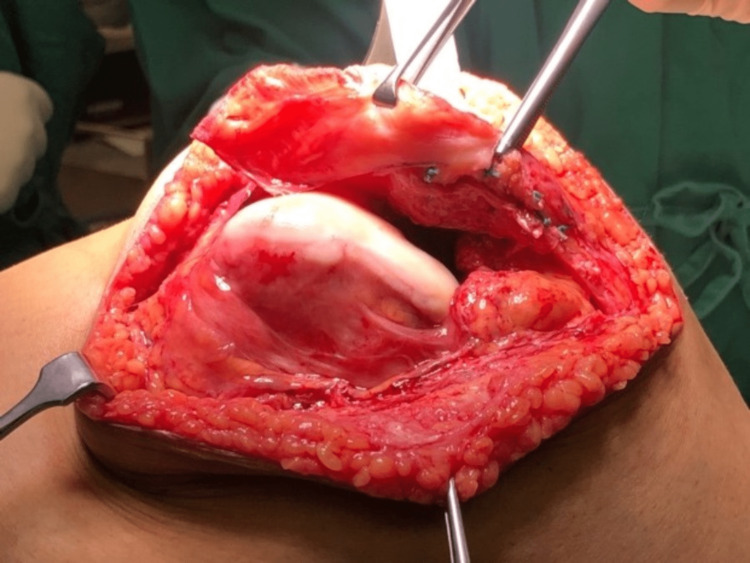
The suture knot should be at the nonarticulating surface of the patella to avoid secondary chondral injury by the Ethibond suture.

We performed an interpositional PFJ arthroplasty with quadriceps tendon procedure on the right knee using a midline and lateral parapatellar approach, as seen in Figure 3. In Figure 4, you can see that we flipped the patella medially to expose the lateral patella facet and quadriceps tendon insertion. To measure the width and length of the lateral and medial patella facet defect, as shown in Figure 5, we took precise measurements. We marked the desired length of the quadriceps tendon that needed to be harvested. It has been observed that the quadriceps tendon is to be taken from the inner lateral region of its attachment on the superior pole of the patella, as highlighted in Figure 6. To prevent overfilling, the procedure using the 15 mm-thick quadriceps tendon illustrated in Figures 7, 8 is performed. As shown in Figure 9, the medial and lateral patella facet is minimally resurfaced with a bone burr. The technique of preparing the patella to be sutured with the quadriceps tendon is illustrated in Figures 10-13. 

Postoperatively, the patient was put on a continuous passive motion (CPM) machine to improve the range of motion. Before discharge, the patient was able to weight bear and her range of motion was 0 - 90 degrees in active and passive motion. Her knee extensor mechanism is intact with power 4. Currently, the patient is post-operation for three and her pain is much reduced. She was referred to physiotherapy for an intensive range of motion exercise and quadriceps muscle strengthening exercise. 

## Discussion

Interpositional arthroplasty for the treatment of tricompartmental knee OA has been described as early 1900. A technique used by Murphy using a pedunculated fascial flap was documented in 1905 and further experiments and studies were done using free single or double fascial flap resulting in various outcomes [7]. In 2007, a study done by Oztuna et al. showed an alternative to patellar resurfacing following total knee replacement with a fascial interposition procedure using the fascial-tendinous part of the quadriceps tendon [8]. The study depends on the theory of the interposition fascia or tissue at the PFJ remained viable and changed to fibrocartilage-like tissue providing a cushioning effect and preventing further damage to the articulating cartilage.

The decision for interpositional arthroplasty of the PFJ was made carefully following a thorough assessment including the specific location of the pain, physical examination, radiological images, and patient expectations. Typical PFJ OA symptoms of medial and lateral patella facet tenderness were elicited in this patient. Radiographical evidence of isolated PFJ OA can be seen in the right knee Merchant view radiograph.

A study done in 2001 showed excessive femoral internal rotation and anteversion as well as tibial external rotation resulted in an increase in lateral patellar tilt and rotation, thus increasing the risk of lateral patellar contact pressure [9]. Concerning our patient, the long leg lower limb radiographical study of the patient ruled out any femoral or tibial component pathology; hence, the non-bony procedure of the PFJ was chosen.

The patellar component was assessed before surgery. Adhered to the principle, the patellar resurfacing procedure required a minimum of 12mm thickness of the patella after resurfacing to avoid fracture [10] and the minimum thickness offered for the patellar button is 9mm. The risk of patella fracture is high as the patient’s patellar thickness is only 12mm. Following this assessment, the patient was not a candidate for patellar resurfacing.

Overstuffing in patellar resurfacing or interpositional arthroplasty was not well documented [11]. Hence, the harvested quadriceps tendon was layered down to the desired thickness of 10mm, restoring 22mm normal patellar thickness in a woman. Range of motion and patellar tracking were assessed after the interposition procedure was done.

This case has a limitation as the follow-up is only three months. The actual outcome needs to be re-evaluated once the soft tissue healed. Intensive physiotherapy is ongoing to restore the preoperative range of motion. We believed that the interpositional arthroplasty in this patient was done as a temporary measure to control the pain before the patient developed full-blown tricompartmental knee OA.

## Conclusions

Resection arthroplasty has historically been the predominant procedure before modern arthroplasty takes place, while interpositional arthroplasty has not been extensively documented. This study presents a detailed step-by-step guide for performing interpositional arthroplasty. Our decision to utilize this technique was influenced by a young patient's unsuitability for patellar resurfacing surgery and the desire to delay total knee arthroplasty. A three-month follow-up revealed significant enhancements in pain management and patient functionality. The author recommends considering interpositional arthroplasty on a case-by-case basis, particularly as a viable option for isolated PFJ OA. Additionally, the author emphasizes the need for further research into treating isolated PFJ OA in younger patients due to its increasing prevalence. The limited treatment options available remain a challenge for individuals with early-onset PFJ OA.
